# Mulching influences pear yield and quality by changing rhizosphere microbial community structure in the arid region of Northwest China

**DOI:** 10.3389/fpls.2025.1633540

**Published:** 2025-09-29

**Authors:** Hongxu Li, Peigen Li, Gang Cao, Mingxin Zhao, Zhiyi Zhu, Yanwei Ma, Wei Wang, Sufang Cao, Yangchun Xu, Caixia Dong

**Affiliations:** ^1^ Institute of Fruit and Floriculture Research, Gansu Academy of Agricultural Sciences, Lanzhou, Gansu, China; ^2^ Jiangsu provincial key lab for solid organic waste utilization, Key lab of organic-based fertilizers of China, Jiangsu Collaborative Innovation Center for Solid Organic Wastes, Educational Ministry Engineering Center of Resource-saving fertilizers, Nanjing Agricultural University, Nanjing, China

**Keywords:** indicator species, drip irrigation, principal coordinate analysis, sugar/acid ratio, pear

## Abstract

**Introduction:**

Mulching is widely adopted in pear orchards to improve soil quality and fruit production, yet its effects on rhizosphere microbial communities and the mechanisms linking soil–microbe interactions to pear yield and quality remain poorly understood.

**Methods:**

A field experiment was conducted in a pear orchard located in the arid region of Northwest China. Three treatments were applied: no mulching (CK), plastic film mulching (FM), and straw mulching (SM). Soil physicochemical properties were analyzed, and rhizosphere microbial community characteristics were assessed using high-throughput sequencing of 16S rRNA and ITS. Network analysis and multivariate statistical approaches were employed to explore microbial community structure, ecological modules, and their relationships with soil properties and fruit traits.

**Results:**

Both FM and SM significantly improved pear yield and fruit quality compared with CK. Principal coordinate analysis showed that mulching significantly altered soil microbial community structure. Proteobacteria and Acidobacteria dominated the bacterial community, while Ascomycota was the predominant fungal phylum. FM increased the abundance of Gram-negative bacteria and reduced Gram-positive groups. Network analysis indicated that FM enhanced ecological modules enriched in indicator species positively correlated with yield and sugar/acid ratio. Soil moisture, nutrient content, and organic matter were identified as major drivers of yield and fruit quality.

**Discussion:**

These findings demonstrate that mulching improves pear yield and quality by modifying soil properties and rhizosphere microbial networks. Plastic film mulching was more effective than straw mulching, further enhancing fruit production by improving soil nutrient content, moisture, and microbial community composition, including the recruitment of functional microbes.

## Highlights

- In Northwest China, both plastic and straw mulching notably enhanced pear yield and quality.
- Mulching significantly altered rhizosphere’s microbial structure, boosting pear performance.
- Mulching improved pear productivity by modulating soil moisture, organic matter, and microbes.

## 1 Introduction

In the face of escalating global climate change and continuous population growth, water scarcity presents a formidable challenge to agriculture in arid and semi-arid regions, particularly in Northwest China, where annual precipitation is typically less than 200–400 mm, potential evaporation exceeds 1500 mm, and large diurnal temperature variations prevail. Consequently, identifying strategies to reduce irrigation water use while maintaining crop yields is essential for the sustainable development of agriculture and the rational utilization of water resources in these areas. Groundcovers, such as living grass cover, straw mulch, and plastic film mulch, play a pivotal role in enhancing crop water use efficiency by mitigating soil erosion and minimizing evaporation. These methods are extensively employed to boost the productivity of dryland agricultural ecosystems. Plastic film mulching, a prevalent method in farmland management, leverages the impermeable nature of plastic films to elevate soil moisture and temperature, thereby enhancing soil fertility, accelerating crop maturity, and increasing yields (Thakur and Kumar, 2020). For instance, Plastic mulch significantly improves soil quality by increasing levels of soil organic matter (SOM) and nutrients, which, in turn, enhances the yield and quality of peaches (Guo et al., 2024). Conversely, straw mulching, another widely utilized agricultural practice, effectively curbs evaporation and augments water infiltration. It also increases loose humus, thus boosting SOM and enhancing soil fertility (Poeplau and Don, 2015; Stefani et al., 2018). Additionally, Straw mulching markedly lowers soil conductivity and nitrate nitrogen (NO_3_^–^N) levels while elevating organic carbon, phosphorus, and potassium (K) contents in the soil, consequently improving both the yield and quality of celery (Zheng et al., 2023). The integration of plastic film mulching with drip irrigation represents an innovative approach that not only reduces evaporation but also fosters favorable water-air and thermal conditions conducive to organic matter decomposition and soil microbial activity. This technology significantly enhances water use efficiency and has gained widespread adoption in arid and semi-arid regions.

The interaction between soil microbes and plants is critically important for agricultural productivity, as microbes play a pivotal role in maintaining the stability of soil ecosystems (Fierer, 2017; Philippot et al., 2024; Saleem et al., 2019). Soil microbes are instrumental in decomposing plant and animal residues, forming SOM and aggregates, which significantly impact soil structure (Xu et al., 2020). Additionally, they enhance soil nutrient availability and cycling (Fernandez et al., 2016). Recent research indicates that mulching profoundly affects the composition and functionality of soil microbial communities. Straw mulching alters the bacterial and fungal community structures in cornfields, enhancing the production of active carbon and nitrogen components and accelerating the carbon and nitrogen cycles (Liu et al., 2023) Similarly, straw mulching fosters the growth of soil fungi and Gram-negative bacteria (Zhao et al., 2016). Plastic film mulching significantly changes the fungal community composition in temperate semi-arid regions (Liu et al., 2012).Furthermore, Plastic film mulching notably reduces soil microbial functional diversity (Xie et al., 2022). Given that soil bacterial and fungal communities may respond differently to various mulching practices, it is essential to explore both the diversity and composition of these microbial communities under different mulching conditions. Such investigations will deepen our understanding of how different mulching strategies influence soil microbial community structures.

While previous studies have highlighted the influence of mulching on fruit tree growth through its impact on soil microbes, the specific mechanisms remain largely unexplored. In the unique climate and soil conditions of Northwest China’s arid regions, soil microorganisms are vital for maintaining soil ecosystem health and achieving high yields and quality in pear orchards. However, detailed research into how mulching impacts soil microbial communities and their interactions with pear trees is sparse. This study aims to conduct a comprehensive evaluation of the effects of mulching—specifically plastic film and straw mulching—on the soil physicochemical properties, microbial community structure, and growth of pear trees in the drip-irrigated arid regions of Northwest China. Network analysis was utilized to investigate the interactions among microbial communities and their correlations with pear yield and quality. By demonstrating the beneficial effects of mulching on soil quality, fruit yield, and quality, this study supports the sustainable management of pear orchards in the arid regions of Northwest China and elucidates the underlying mechanisms. The main aim of this study was to evaluate the effects of different mulching practices (plastic film mulching, straw mulching, and no mulching) under drip irrigation on pear yield, fruit quality, and rhizosphere microbial community structure in the arid regions of Northwest China. By linking soil physicochemical properties with microbial community dynamics, we sought to reveal the mechanisms by which mulching influences pear performance and to provide a scientific basis for sustainable orchard management in dryland areas.

## 2 Materials and methods

### 2.1 Experimental site

**Figure 1:**
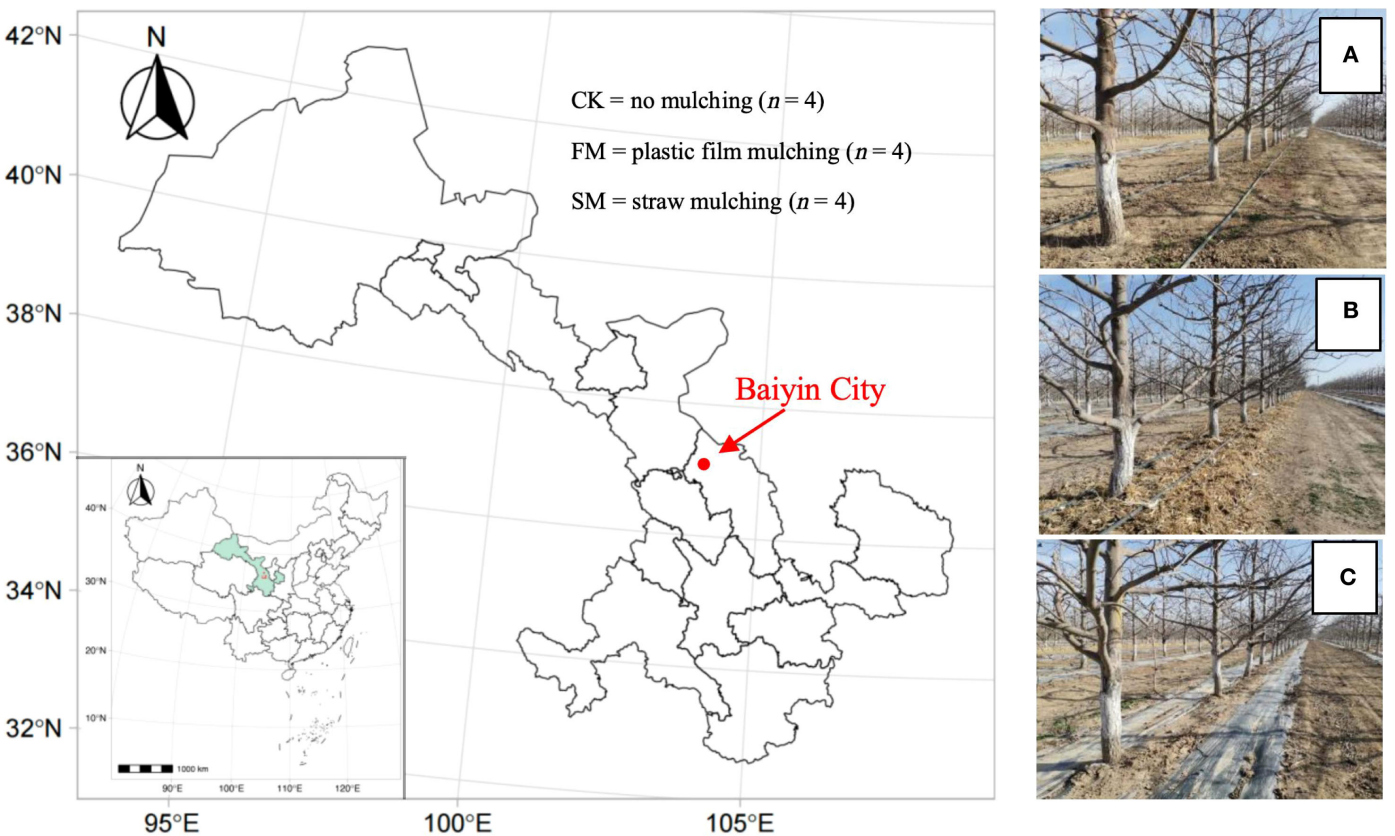
Map showing the experimental site (indicated by the red dot) in Jingtai County, Baiyin City, Gansu Province, China (left) and photos showing the three treatments of no mulching **(A)**, straw mulching **(B)**, and plastic film mulching **(C)**.

The study was conducted in Jingtai County, Baiyin City, located in Gansu Province (36°43’ N, 103°33’ E; **Figure 1**). Monthly mean temperature, precipitation, and other climatic parameters during the experimental period are presented in **Supplementary Table S7**. The soil type is calcareous. Key physicochemical properties of the soil within the top 20 cm include a pH of 8.6, organic matter content of 5.53 g/kg, ammonium nitrogen (NH_4_^+^-N) concentration of 2.35 mg/kg, NO_3_^–^N concentration of 6.06 mg/kg, available phosphorus at 46.61 mg/kg, available K at 258.79 mg/kg, and available iron (Fe) at 4.11 mg/kg.

### 2.2 Experimental materials and design

The pear (*Pyrus communis* L.) variety Huangguan was planted in the orchard with an intra-row plant spacing of 2 m and an inter-row spacing of 4 m. In 2015 when the trees were two years old, three treatments were set up: plastic film mulching (FM), straw mulching (SM), and no mulching (CK; **Figures 1a–c**). For the FM treatment, 0.012 mm-thick black polyvinyl chloride film was used as mulch to cover a 2.4 m-wide area under canopy of each row. The plastic films were removed after harvest and replaced with new ones each April (spring) before budbreak. For the SM treatment, corn straw, which had been chipped and baled, was used as mulch to cover a 2.4 m-wide area under canopy of each row, with a thickness of about 18 cm. Each October (after harvest), additional straw was applied to restore the thickness to ~18 cm, as the previous layer gradually collapsed and decomposed, rather than being completely replaced. For the CK treatment, weeding was performed after irrigation, with a total of four times a year. Every autumn, a trench (40 cm wide, 40 cm deep, 30 cm inward from the outskirt of canopy projection) was dug along each row of trees, and approximately 36 kg of composed sheep manure was applied per tree per year. In addition, 0.24 kg of monoammonium phosphate was applied per tree per year via fertigation using the drip irrigation system. The basic physicochemical properties of the sheep manure are provided in **Supplementary Table S1**.

### 2.3 Sampling

For each treatment, four adjacent trees of similar size were pooled together to form one biological replicate. In total, four replicates (each consisting of four pooled trees, i.e., 16 trees per treatment) were established. Pear fruit samples were collected at harvest on September 15, 2022. Five fruits were randomly collected from different directions of each tree and transferred to the laboratory for determination of fruit quality-related properties.

Soil samples were collected after fruit harvest. Under each tree where pear fruit samples were taken, four points 30 cm inward from the outskirt of canopy projection (approximately 1 m from the trunk) and away from where fertilizers were applied were randomly selected. Soil samples were taken from the 15–20 cm layer at the four points and combined to make a composite sample of approximately 400 g (100 g from each point). The soil samples were air-dried before analyzed for physicochemical properties. Additionally, rhizosphere soil adhering to root surface was gently sampled, put into 1.5-ml centrifuge tubes, transferred to the laboratory in a cooler, and stored at -80 °C for later use.

### 2.4 Laboratory measurements and analyses

Total microbial DNA was extracted using a soil DNA extraction kit (Omega Bio-Tek, Norcross, GA, USA). The concentration and purity of DNA were assessed using a UV-Vis spectrophotometer (NanoDrop 2000, Thermo Scientific, Wilmington, USA). The integrity of DNA was evaluated using 1% agarose gel electrophoresis.

The primer set 341F (5’-CCTAYGGGRBGCASCAG-3’)/806R (5’-GGACTACNNGGGTATCTAAT-3’) was used to amplify the V3–V4 region of bacterial 16S rRNA gene using PCR. The primer set ITS1F (5’-CTTGGTCATTTAGAGGAAGTAA-3’)/ITS2R (5’-GCTGCGTTCTTCATCGATGC-3’) was used to amplify the ITS1 region of internal transcribed spacer (ITS) in fungi. The PCR program was set as follows: initial denaturation at 95°C for 3 min, 27 (bacterial 16S rRNA) or 35 cycles (fungal ITS) of denaturation at 95 °C for 30 s, annealing at 55 °C for 30 s, and extension at 72 °C for 45 s, and final extension at 72 °C for 10 min. The PCR products were then purified, quantified, and normalized to create a sequencing library, which was sequenced by Shanghai Biozeron Biotechnology Co., Ltd. using the Illumina NovaSeq PE250 platform.

Fruit samples were weighed. Fruit length and diameter were measured using a vernier caliper (500-196-30, Mitutoyo, Kanagawa, Japan). Soluble solid content was measured using a refractometer (ATAGO-1, ATAGO, Tokyo, Japan). Fruit firmness was measured with a penetrometer (FT327, Shanghai Precision and Scientific Instrument Co., Shanghai, China). Titratable acidity (TA) of pear juice was determined by neutralization titration with standardized 0.1 mol L^-1^ NaOH solution to a phenolphthalein endpoint (pH ≈ 8.2), and expressed as malic acid equivalents according to Bao (2000). Soluble sugar content was determined by the anthrone-sulfuric acid colorimetric method (Yemm and Willis, 1954).Soluble sugars (sorbitol, fructose, glucose) were analyzed by HPLC-RI on a CARBOSep CHO-620 Ca column with water as the mobile phase, while organic acids (malic, citric) were determined by HPLC-UV on a ZORBAX Eclipse XDB-C18 column with phosphate buffer (pH 2.6) as the mobile phase. External calibration with authentic standards was used for quantification, following published methods (Filip et al., 2016).

Soil available nitrogen was determined by the alkali diffusion-titration method, available phosphorus by the molybdenum antimony anti-colorimetric method after 0.5 M NaHCO_3_ extraction, and available potassium by 1 M NH_4_OAc extraction followed by flame photometry (Bao, 2000). Soil micronutrients were extracted with diethylenetriaminepentaacetic acid (DTPA). Briefly, 10 g of air-dried soil was added to 20 ml of DTPA solution (0.005 M DTPA, 0.01 M CaCl2, 0.1 M triethanolamine, pH 7.3), shaken at room temperature for 2 h, and filtered. The concentrations of Fe, manganese (Mn), copper (Cu), and zinc (Zn) in filtrate were then measured using inductively coupled plasma-optical emission spectrometry (ICP-OES, Optima 7300 DV, PerkinElmer).

### 2.5 Statistical and analysis

Amplicon data analysis was performed using the EasyAmplicon v1.18 pipeline (Liu et al., 2023). Raw sequencing generated 26,466–70,306 paired-end reads per sample (average ~52,000, 250 bp). Rarefaction curves (**Supplementary Figure S4**) approached saturation and Good’s coverage exceeded 0.97, indicating sufficient sequencing depth.Specifically, paired-end reads were merged using the fastq_mergepairs command, quality control was performed using the fastx_filter command, and dereplication was performed using the derep_fulllength command in VSEARCH v2.22 (Rognes et al., 2016). Non-redundant sequences were denoised into amplicon sequence variants using the unoise command in USEARCH v10.0 (Edgar, 2010), with a minimum abundance threshold of 10 (minsize = 10) to denoise and infer ASVs, while filtering chimeras. The uchime_ref command in VSEARCH was then used to align feature sequences to the SILVA database for further chimera removal (Quast et al., 2013; release 123). Next, a feature table was generated using the usearch_global command in VSEARCH. Taxonomic assignment of feature sequences was performed using the sintax algorithm in USEARCH and the RDP training set v18 (Cole et al., 2014).

Network construction and module detection. Co-occurrence networks were built on genus-level relative abundance tables using Spearman correlations; edges were retained when p ≥ 0.6. The resulting undirected graphs were modularized with greedy modularity optimization on the as.undirected network; node coordinates were computed by the Fruchterman–Reingold layout.”Indicator species (taxa)”for each treatment were identified using indicspecies::multipatt with func = “r.g” and 999 permutations; taxa with p < 0.05 were retained as indicators and then annotated back to genera. To increase rigor, p-values were additionally FDR-adjusted (Benjamini–Hochberg) and only taxa with q < 0.05 were considered significant in the final reporting.

Random forest analysis combined with Spearman analysis was used not only to analyze the contribution of dominant rhizosphere groups (at the genus level) to bacterial and fungal communities on root growth but also to assess the important factors affecting above-ground pear growth under different mulching treatments (R v4.2.2, ‘randomForest’ and ‘rfPermute’ packages). Diversity analysis was conducted using the vegan v2.6–4 package in R v4.2.2 (Oksanen et al., 2007), and data visualization was performed using the ggplot2 v3.4.1 package (Wickham, 2016).

## 3 Results

### 3.1 Pear fruit yield and quality

**Figure 2:**
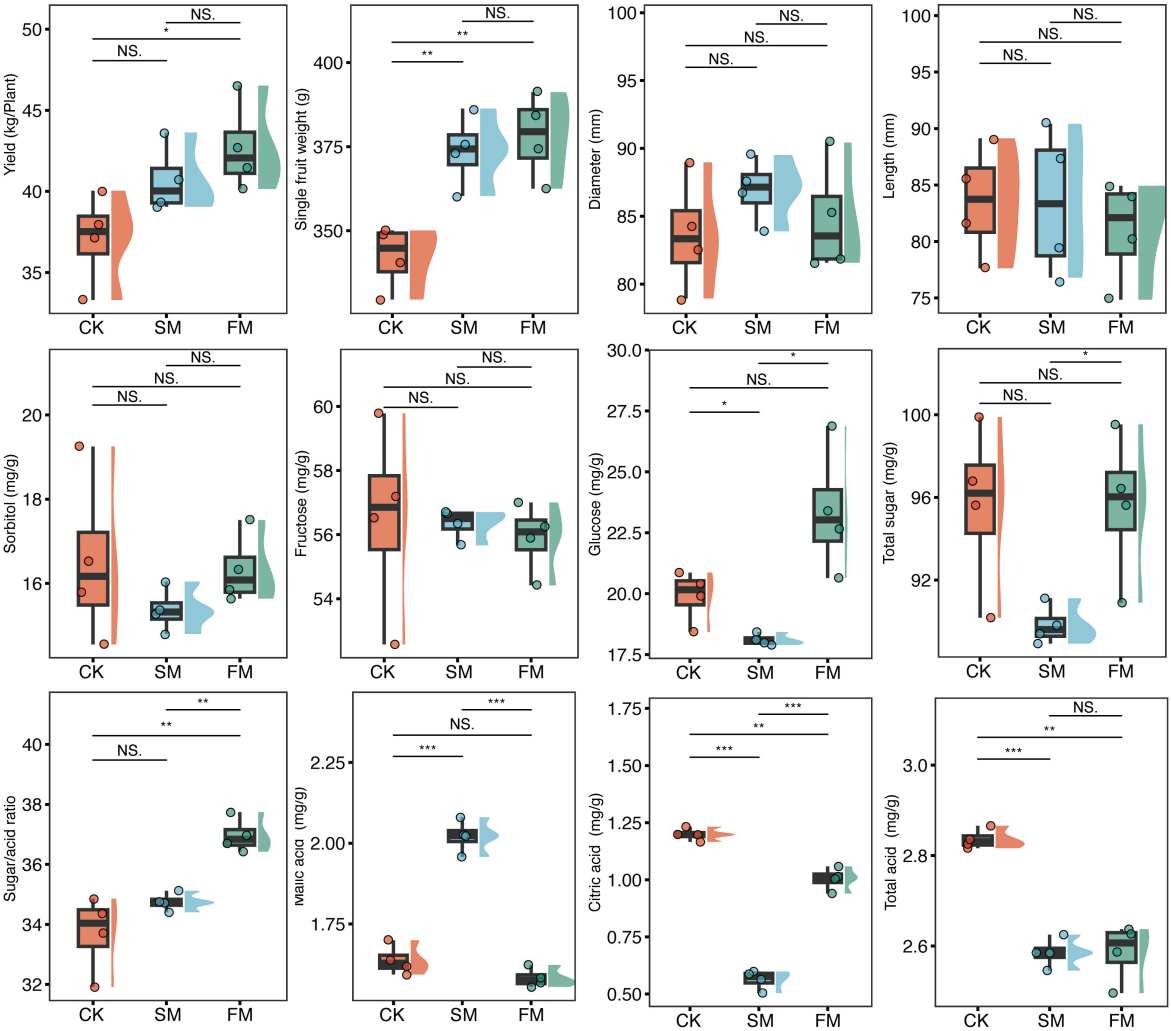
Pear fruit yield, single fruit weight, diameter, length, sorbitol content, fructose content, glucose content, total sugar content, sugar/acid ratio, malic acid content, citric acid content, and total acid content in the different treatments. ***p < 0.001; ** p < 0.01; *p < 0.01; p < 0.05; ns: p > 0.05. CK, no mulching; FM, plastic film mulching; SM, straw mulching.

Pear yield was significantly influenced by mulching practices (*p* = 0.037; **Figure 2**). Relative to CK, yields increased by 15.1% in plots of FM (*p* = 0.041) and by 9.67% in those of SM (*p* = 0.087). Principal coordinate analysis (PCoA) delineated three distinct clusters representing the quality characteristics of pear fruit across different treatments (PERMANOVA: *R* ^2^ = 0.44, *p* < 0.01; **Supplementary Figure S1**). The total organic acid content in both FM and SM was significantly reduced by 8.79% and 8.84%, respectively, compared to CK (**Figure 2**). Notably, the citric acid content decreased by 16.2% in FM and by 53.0% in SM. Additionally, SM significantly increased the malic acid content to 1.23 times that of CK. Conversely, the total sugar content in SM was significantly reduced by 6.07% compared to CK, with no significant difference observed between FM and CK. There were also no significant differences in fructose and sorbitol contents across the treatments.

### 3.2 Rhizosphere soil physicochemical properties

**Figure 3:**
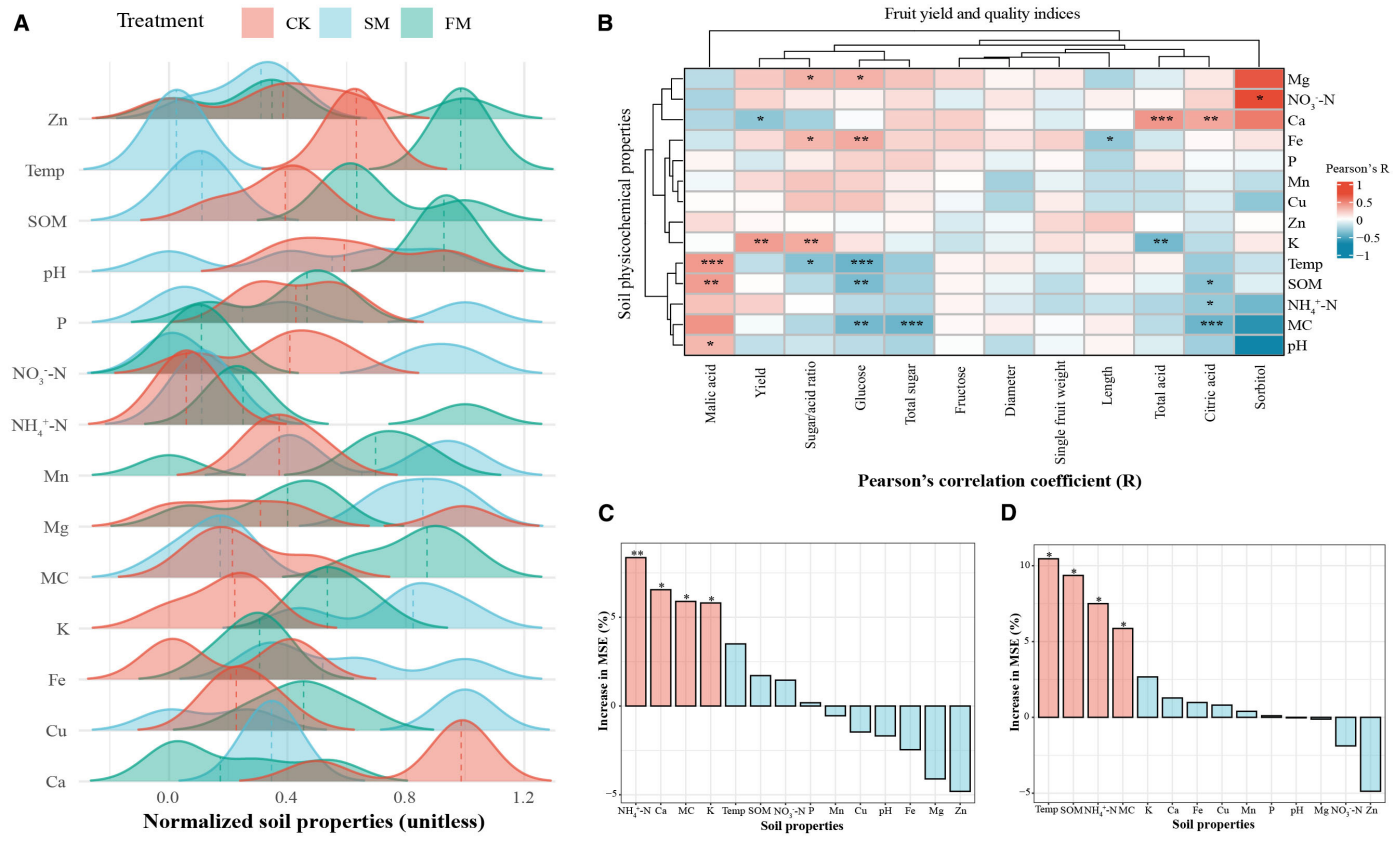
Soil physicochemical properties and their influences on pear fruit yield and quality in different mulching treatments. **(A)** Changing trends of normalized soil physicochemical properties. **(B)** Correlations between fruit yield and quality indices and soil physicochemical properties. (***p < 0.001; *p < 0.01; p < 0.05). **(C)** Contributions of soil physicochemical properties to fruit yield based on the random forest model. **(D)** Contributions of soil physicochemical properties to fruit quality based on the random forest model. The data in the bar charts had been standardized using the Z-score. Red bars indicate significant changes (*p < 0.05, **p < 0.01), whereas blue bars indicate non-significant changes. CK, no mulching; FM, plastic film mulching; SM, straw mulching. Temp = temperature; SOM = soil organic matter; MC = moisture content.

In this study, the FM treatment enhanced soil moisture content (MC) by 25.8%, while SM decreased it by 4.8% (**Figure 3A**, **Supplementary Table S2**). Similarly, FM significantly raised soil temperature by 12.1%, whereas SM reduced it by 20.1%. Additionally, FM boosted SOM content by 15.5%, while SM reduced it by 11.8%. Relative to CK, FM significantly increased the levels of NH_4_^+^-N and K by 35.5% and 15.0%, respectively, whereas SM showed more modest increases (*p* < 0.05) of 5.4% and 23.7%, respectively. In terms of micronutrients, FM treatment increased soil Fe, Mn, Cu, and Zn contents by 7.3%, 6.3%, 6.1%, and 7.7%,respectively. Conversely, SM led to increases in soil Fe, Mn, and Cu contents by 40.1%, 10.2%, and 9.5%, respectively, but saw a decrease in Zn content by 6.9%. Compared to CK, FM also led to significant reductions in soil NO_3_^–^N and calcium (Ca) contents by 46.2% and 22.7%, respectively, while SM increased NO_3_^–^N and magnesium (Mg) contents by 16.2% and 10.7%, respectively. Correlation analysis revealed that soil K was positively correlated with pear yield (*p* < 0.01; **Figure 3B**), while Ca showed a negative correlation (*p* < 0.05). Soil K, Ca, and Mg contents were positively correlated with the pear sugar/acid ratio, whereas soil temperature showed a negative correlation (*p* < 0.05). Furthermore, soil NO_3_^–^N content was positively associated with pear fruit sorbitol content (*p* < 0.05), but soil SOM (*p* < 0.01), temperature (*p* < 0.001), and MC (*p* < 0.01) were negatively correlated with pear fruit glucose content. Available soil Fe (*p* < 0.01) and Mg (*p* < 0.05) were positively correlated with fruit glucose content, while soil moisture negatively impacted the total sugar content in pear fruit (*p* < 0.001). Soil Ca content was positively correlated with pear total acid content (*p* < 0.001), while K content was negatively correlated (*p* < 0.01). Soil pH (*p* < 0.05), temperature (*p* < 0.001), and SOM (*p* < 0.01) were found to be positively correlated with fruit malic acid content. Random forest prediction indicated that soil physicochemical properties contributed 27% to fruit yield and 32% to fruit quality (**Figures 3C, D**). This suggests that mulching significantly enhances pear fruit yield and quality by modulating soil physicochemical properties, thereby influencing the availability and balance of essential nutrients and conditions favorable for optimal fruit development.

### 3.3 Rhizosphere microbial community composition and phenotype

**Figure 4:**
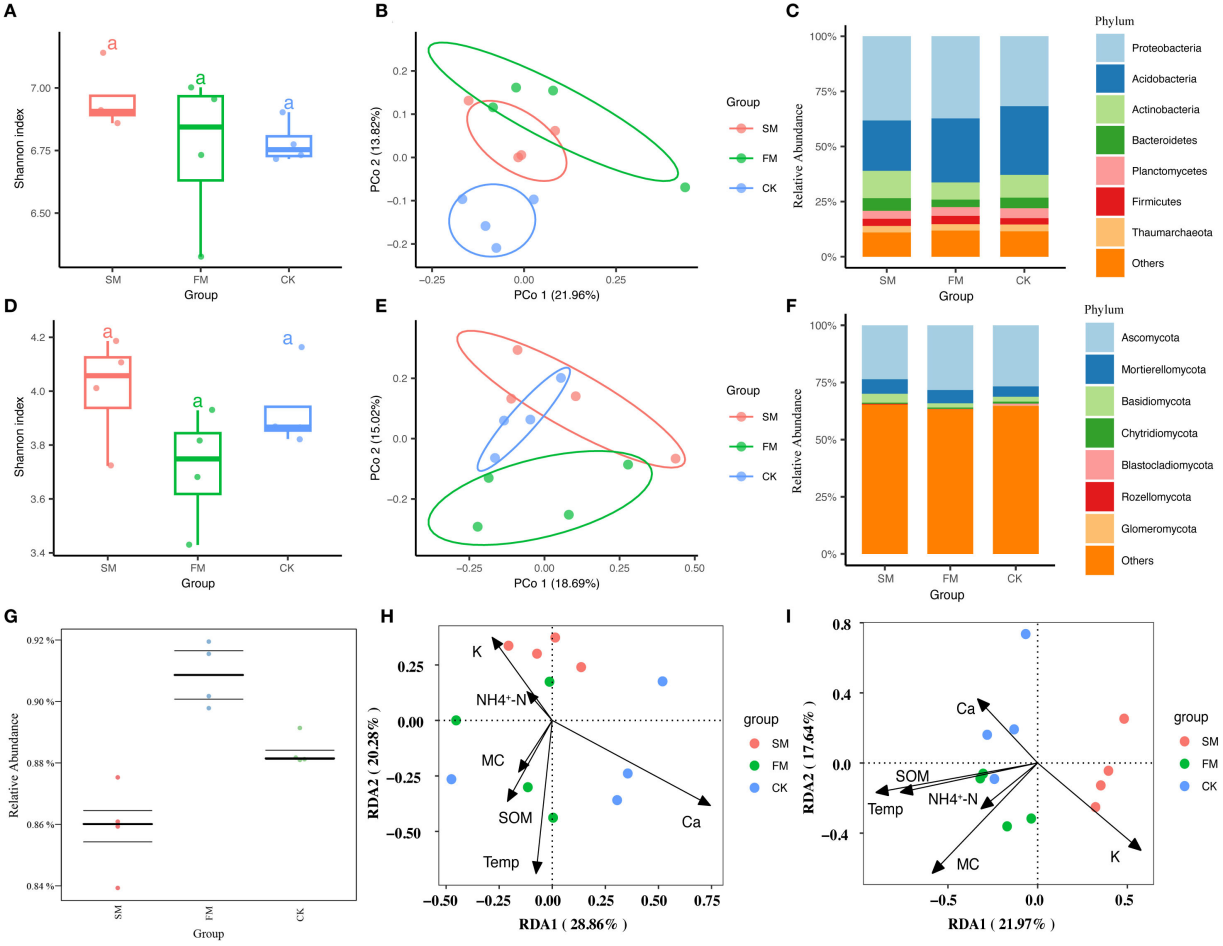
Differences in rhizosphere bacteria and fungi in different treatments. **(A)** Bacterial community α-diversity (Shannon diversity index) in different treatments. **(B)** Bacterial community β-diversity (based on Bray-Curtis distance) in different treatments. **(C)** Relative abundances of the seven most dominant bacterial phyla in different treatments. **(D)** Fungal community α-diversity (Shannon diversity index) in different treatments. **(E)** Fungal community β-diversity (based on Bray-Curtis distance) in different treatments. **(F)** Relative abundances of the seven most abundant fungal phyla in different treatments. **(G)** BugBase predicted abundances of Gram-negative bacteria in different treatments. **(H)** Redundancy analysis (RDA) of the relationships between rhizosphere soil bacterial communities and soil physicochemical properties in different treatments. **(I)** RDA of the relationships between rhizosphere soil fungal communities and soil physicochemical properties in different treatments.

The Shannon diversity indices for rhizosphere bacteria and fungi did not show significant differences between treatments (**Figures 4A, D**). However, PCoA results revealed that mulching significantly affected the community compositions of both rhizosphere bacteria and fungi (**Figures 4B, E**), as confirmed by PERMANOVA tests (bacterial community:F=1.15, R^2^ = 0.20, p=0.011; fungi community: F = 0.62, R^2^ = 0.12, p=0.037). Specifically, bacterial communities exhibited more distinct separation along the second axis, with the first two axes accounting for 35.78% of the total variance—21.96% from PCo1 and 13.82% from PCo2. Similarly, fungal communities showed clear distinctions along the second axis, with the first two axes explaining 33.71% of the total variance—18.69% attributed to PCo1 and 15.02% to PCo2.

At the phylum level, the bacterial community in the rhizosphere was predominantly composed of Proteobacteria, followed by Acidobacteria, Actinobacteria, Bacteroidetes, Planctomycetes, Firmicutes, and Thaumarchaeota (**Figure 4C**). These seven phyla collectively constituted between 90.7% and 91.8% of the total bacterial relative abundance, significantly shaping the soil bacterial community composition in the pear orchard. Proteobacteria and Acidobacteria were notably the most dominant, representing 61.7% to 64.9% of the total bacterial relative abundance. Relative to CK, both SM and FM treatments increased the relative abundances of Proteobacteria and Firmicutes, while decreasing those of Acidobacteria and Planctomycetes (**Supplementary Table S3**).The fungal community was led by Ascomycota, followed by Mortierellomycota, Basidiomycota, Chytridiomycota, Blastocladiomycota, Rozellomycota, and Glomeromycota (**Figure 4F**). These seven phyla accounted for 46.4% to 49.5% of the total fungal relative abundance. Ascomycota and Mortierellomycota were the most dominant phyla, comprising 30.2% to 46.3% of the total fungal relative abundance. Compared to CK, the SM and FM treatments resulted in increased relative abundances of Mortierellomycota and Rozellomycota in the rhizosphere, while those of Chytridiomycota, Blastocladiomycota, and Glomeromycota decreased (**Supplementary Table S4**).

The correlation analysis between the rhizosphere microbial community composition and environmental factors demonstrated significant correlations of MC and Ca with the microbial community composition (**Supplementary Figure S2**). Redundancy analysis (RDA) provided further insight, revealing that RDA1 and RDA2 together accounted for 49.14% of the variance in the bacterial community and 39.61% of the variance in the fungal community (**Figures 4H, I**). Specifically, RDA1 distinctly separated CK from the mulching treatments (FM and SM) in terms of bacterial community composition, while RDA2 did the same for the fungal community. The main soil physicochemical properties driving differences in bacterial community structure among the treatments were MC, temperature, and SOM. In contrast, the variance in fungal community structure was primarily influenced by MC, temperature, NH_4_^+^-N, and SOM. These findings underscore the significant impact of specific soil properties on the composition and structure of microbial communities in the rhizosphere.

BugBase was utilized to predict bacterial phenotypes, revealing significant differences in the abundances of both Gram-positive and Gram-negative bacteria between treatments (*p* < 0.05). Notably, the abundance of Gram-negative bacteria was significantly higher in the FM treatment (*p* < 0.05), while Gram-positive bacteria were significantly reduced in FM (**Figure 4G**, *p* < 0.05). Specifically, FM significantly increased the relative abundances of Proteobacteria, Gemmatimonadetes, and Bacteroidetes, which are Gram-negative taxa known to be closely associated with soil carbon and nutrient cycling. These findings underscore the profound impact of mulching on bacterial phenotypes within the soil. Additionally, regression analysis indicated a significant negative correlation between soil MC and the abundance of Gram-positive bacteria across all treatments (**Supplementary Figures S3**, *p* < 0.01). This analysis highlights the critical influence of soil moisture on the microbial dynamics within the rhizosphere, particularly concerning Gram-positive bacterial populations.

### 3.4 Rhizosphere bacterial network structure

**Figure 5:**
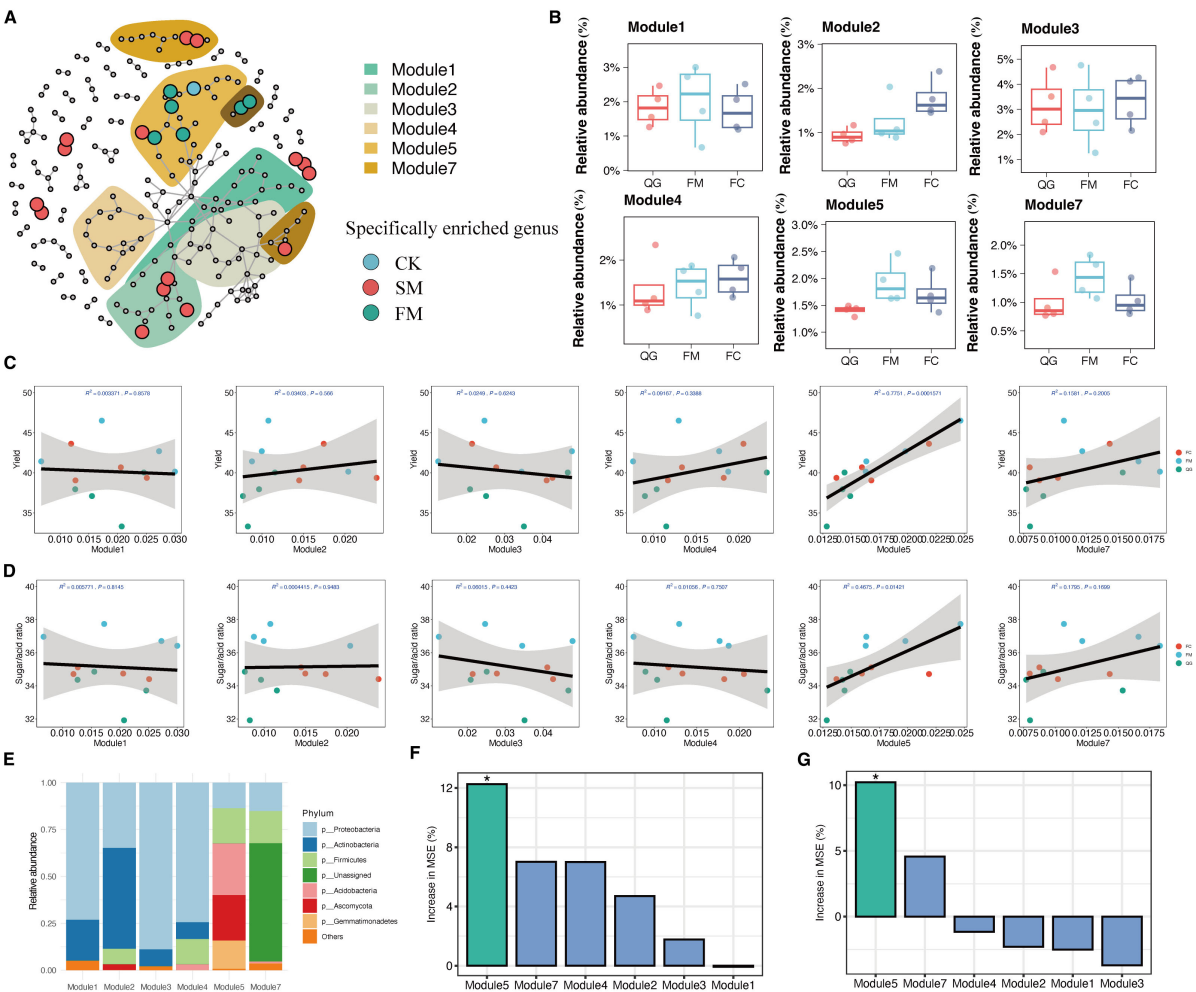
Microbial co-occurrence network and the correlations between enriched modules and pear yield. **(A)** Co-occurrence network showing significant correlations between bacterial communities, functional profiles, and yield indicators (R > 0.9, p < 0.05). The shaded areas (modules) indicate the ecology (nodes > 9) of co-occurrence network indicators and the cumulative abundance of each ecological cluster module. Significant differences in cumulative abundance between treatments are shown. **(B)** Relative abundances of the ecological modules in different treatments. **(C)** Correlations between pear yield and the cumulative relative abundances of ecological modules. **(D)** Correlations between pear quality and the cumulative relative abundances of ecological modules. **(E)** The seven most abundant phyla in the rhizosphere microbial communities within different modules. **(F)** Contributions of soil microbial modules to changes in fruit yield based on the random forest model. **(G)** Contributions of soil microbial modules to changes in fruit quality based on the random forest model. The data had been standardized by Z-score. Green bars indicate significant differences (*p < 0.05), whereas blue bars indicate non-significant difference. The data of each module in the histogram are standardized by Z-score.

Mulching had a pronounced impact on the rhizosphere bacterial ecological network, leading to significant variations in the distribution patterns of indicator species within this network (**Figure 5**). The bacterial co-occurrence network comprised 112 nodes, of which 60 were identified as indicator species nodes. These included 16 unique to CK, 15 unique to FM, and 29 unique to SM (**Figure 5a**, **Supplementary Table S5**). Differences in microbial ecological groups were also evident between treatments (**Figure 5**). Specifically, within the bacterial community of the FM treatment, distinct ecological modules, M5 and M7, emerged, containing indicator species unique to FM (**Figure 5A**). Conversely, ecological modules M2 and M4 included indicator species exclusive to SM, while M3 was primarily composed of indicator species specific to CK. Modules M1, M2, M4, M5, and M7 formed the core components of the network, with a clear delineation between M5 and M7, which predominantly contained FM-specific indicator species, and M2 and M4, which mainly included SM-specific indicator species. This structural distinction underscores the influence of mulching type on the organization and function of microbial communities within the rhizosphere.

The sensitivity of module members to specific treatments and their distribution in the network provided insights into the factors driving differences in bacterial communities, as observed in the PCoA ordination (**Figures 4B, E**). Notably, the ecological modules M5 (*p* < 0.001) displayed significant variations with mulching treatments and showed distinct responses (**Figure 5B**). In these modules, bacterial species in the FM treatment exhibited higher abundances compared to CK and SM (*p* < 0.05), while no significant differences were observed between CK and SM (HSD test: *p* > 0.05; **Figure 5B**). Correlation analysis further demonstrated a significant positive correlation between the abundance of ecological module M5 and pear yield (*R* ^2^ = 0.78, *p* < 0.001), whereas no significant correlations were found between other modules and pear yield (**Figure 5C**). Similarly, the abundance of ecological module M5 was positively correlated with pear fruit quality (*R* ^2^ = 0.56, *p* < 0.001), but other modules showed no significant correlations with fruit quality (**Figure 5D**). This suggests that mulching can modulate the co-occurrence patterns of ecological taxonomies, thereby enhancing pear yield through the increased relative abundances of certain ecological modules. Taxonomic analysis indicated that module M2 was primarily composed of Actinobacteria, whereas M5 consisted mainly of Proteobacteria, Acidobacteria, Gemmatimonadetes, and Firmicutes (**Figure 5E**, **Supplementary Table S6**). Random forest prediction indicated that the microbial modules contributed 30% and 15% to fruit yield and quality, respectively, highlighting the potential of mulching to improve pear yield and quality by altering microbial community structures (**Figures 5F, G**).

### 3.5 Overall influence of mulching on pear yield and quality

**Figure 6:**
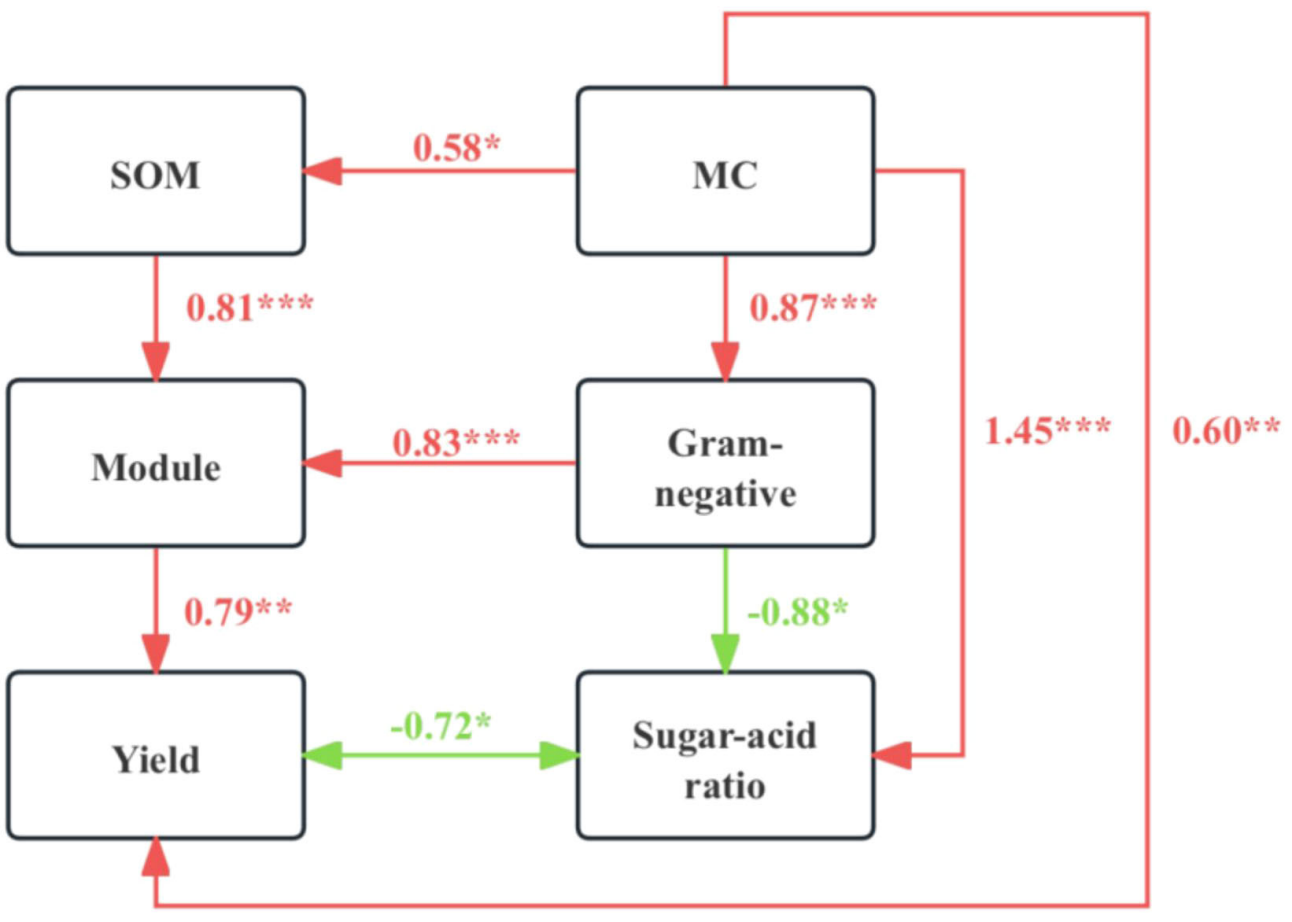
Structural equation model (SEM) of the direct and indirect effects of mulching on pear yield and quality (indicated by the sugar/acid ratio). The module here refers to ecological module5, which was significantly associated with pear yield and sugar/acid ratio. *, p < 0.05; **, p < 0.01; ***, p < 0.001.

The sugar/acid ratio is a critical indicator of pear fruit quality. Significant differences were observed in pear yield, sugar/acid ratio, and their correlations with the abiotic and biotic properties of rhizosphere soil across different mulching treatments. Structural equation modeling (SEM) was utilized to examine how mulching impacts pear yield and quality through direct and indirect relationships involving soil physicochemical and microbial properties. The SEM model demonstrated a reasonable fit and effectively elucidated the variations in pear yield and sugar/acid ratio (**Figure 6**). In this model, soil MC had a positive impact on SOM (*r* = 0.58, *p* = 0.014) and Gram-negative bacteria (*r* = 0.87, *p* < 0.001). Gram-negative bacteria, in turn, positively influenced the soil microbial ecological module (*r* = 0.83, *p* < 0.001). Both the soil ecological module and MC had a positive effect on pear yield (*r* = 0.79, *p* = 0.003 and *r* = 0.60, *p* < 0.01, respectively). Conversely, Gram-negative bacteria and soil MC had negative (*r* = -0.88, *p* = 0.013) and positive (*r* = 1.45, *p* < 0.001) impacts on the sugar/acid ratio of pear fruit, respectively. In summary, mulching significantly enhanced pear yield and quality by modulating soil physicochemical and microbial properties.

## 4 Discussion

### 4.1 The effects of mulching on pear yield and quality in arid regions

Soil quality is pivotal for achieving stable, high crop yields and superior fruit quality. In this study, which employed drip irrigation, both FM and (SM were shown to enhance pear yield and quality (**Figure 2**). This finding aligns with (Chen et al., 2014), who reported that, compared to no mulching, both straw mulching and fresh grass mulching significantly improved apple yield, fruit firmness, and sugar content. Similarly, Various mulching treatments significantly affected grape yield and quality, with plastic film mulching notably increasing the levels of soluble sugars, total phenols, and anthocyanins in grapes (Jabran et al., 2015). In this study, both plastic film and straw mulches exerted positive influences on pear yield and quality. Relative to CK, FM and SM increased pear yield by approximately 15.1% and 9.67%, respectively. This enhancement is likely attributable to mulching improving the stress tolerance of fruit trees, as it is well-documented that mulching boosts fruit firmness, thereby reducing mechanical damage from fruit drop and subsequent yield losses. Additionally, mulching has been shown to increase the net photosynthetic rate of fruit trees, potentially due to enhanced nitrogen use efficiency in leaves and sustained leaf vitality during critical growth stages, which are crucial for photosynthesis during fruit enlargement and maturation (Niu et al., 2020). Furthermore, mulching not only boosts fruit yield but also effectively improves the fruit sugar/acid ratio by moderating soil temperature and reducing ground radiation. In particular, FM was especially effective in increasing the sugar/acid ratio of pear fruit. Notably, SM slightly reduced total soluble sugars (**Figure 2**)—likely due to cooler soil (Demo and Bogale, 2024; Liang et al., 2025), microbial carbon competition during straw decomposition (Mo et al., 2021), and higher respiratory consumption (Dang et al., 2024)—so the higher sugar/acid ratio under SM was mainly driven by decreased organic acids rather than sugar increases. Both FM and SM decreased the total organic acid content, notably citric acid, thereby enhancing fruit flavor and quality (**Figure 2**).

Mulching enhances pear tree growth and fruit quality not only by directly improving soil physicochemical properties but also by indirectly changing the rhizosphere microbial community structure and function (Steinmetz et al., 2016; Sun et al., 2020; Yao et al., 2005). The primary objectives of mulching are to regulate soil temperature and to increase soil moisture, both of which are crucial for orchard production systems in semi-arid areas. In this study, both FM and SM effectively increased soil MC, as demonstrated in **Figure 3a**, **Supplementary Table S2**. Compared to no mulching, straw mulching typically results in higher soil moisture levels, likely because straw increases soil surface roughness, thereby reducing runoff and enhancing rainwater infiltration (Basche et al., 2016; Prosdocimi et al., 2016). Conversely, plastic film mulch reduces soil water evaporation due to its impermeability, subsequently increasing soil water availability (Kader et al., 2017; Saglam et al., 2017). The use of plastic mulch not only enhances soil moisture utilization but also increases tomato yield and reduces soil water consumption (Dai et al., 2024). However, it is important to note that soil water retention capacity can decrease gradually with the accumulation of plastic film residues in the soil (Wang et al., 2020a). Residual film fragments may further degrade into microplastics, which alter soil structure, reduce porosity, and impair microbial habitats (Qi et al., 2020). The recovery and recycling of residual film are labor-intensive and costly, especially in perennial orchards (Lodolini et al., 2024). To mitigate such risks, biodegradable mulching films are being promoted as a sustainable alternative, providing similar agronomic benefits while reducing long-term ecological impacts (Park et al., 2025).Additionally, mulching significantly affects soil temperature. In this study, the soil temperature under FM was significantly higher than that in CK, whereas the temperature under SM was significantly lower than that in CK (**Figure 3a**, **Supplementary Table S2**), aligning with findings by Zhang et al. (2017). The reduction in soil temperature under straw mulching is primarily due to the reduced solar energy absorption by the soil. In contrast, plastic film mulch absorbs solar radiation, leading to an increase in soil temperature, particularly noticeable during the summer months (Kader et al., 2017).

In this study, relative to the control with no mulching (CK), both mulching treatments notably enhanced soil fertility, albeit to different extents, significantly impacting pear fruit yield and quality (**Figure 3B**). The FM treatment significantly increased the soil contents of Fe, Ca, and Mg, whereas the SM treatment had no significant impact on these properties. However, both mulching treatments substantially increased SOM content compared to CK. Organic mulching contributes organic carbon to the soil (Sun et al., 2021), thereby effectively boosting SOM content, a crucial indicator of soil fertility and health. Contrary to findings that reported a significant reduction in soil organic matter (SOM) under plastic mulch (Zhang et al., 2023), this study observed a significant increase in SOM with the FM treatment. This discrepancy may be attributed to the specific management conditions applied here: plastic film was combined with subsurface drip irrigation and organic manure application in an arid region. Such practices maintain soil moisture beneath the mulch, reduce organic matter mineralization, and promote the stabilization of microbial residues, while external organic inputs further support SOM accumulation (Yan et al., 2025; Tan et al., 2024). Furthermore, the FM treatment significantly altered the relative abundances of Gram-negative bacteria in the rhizosphere (**Figure 4G**). These microorganisms are crucial in the accumulation and transformation of organic matter, highlighting the complex interactions between mulching practices and microbial dynamics in influencing soil fertility.

### 4.2 Effects of mulching on rhizosphere microbial diversity and functional communities

Soil management practices, such as mulching, profoundly impact rhizosphere microbial diversity and functional communities. In this study, while no significant difference in microbial alpha diversity between treatments was observed (**Figures 4A, D**), the SM treatment was noted to enhance microbial diversity relative to CK, aligning with findings from previous research. PCoA effectively distinguished the various treatments along the PCo2 axis, underscoring the substantial influence of mulching on the structure of rhizosphere microbial communities (**Figures 4B, E**). Soil microorganisms are highly responsive to alterations in soil properties such as SOM and soil water availability, which are affected by agricultural management practices (Wang et al., 2020b). Thus, mulching can modify the composition and diversity of microbial communities by influencing the soil physicochemical environment(**Figures 4H, I**). In comparison to CK, the FM treatment showed an increase in the relative abundances of Proteobacteria and Firmicutes, while those of Acidobacteria, Actinobacteria, and Bacteroidetes decreased (**Figure 4C**; **Supplementary Table S3**). This pattern is consistent with the results (Chen et al., 2014). Notably, Proteobacteria and Firmicutes are critical for enhancing both yield and quality of pear (Jiang et al., 2021). The enrichment of Mortierellomycota under SM is consistent with their role as saprotrophic fungi utilizing straw-derived carbon. Members of this group are reported to decompose cellulose, contribute to nutrient cycling, and in some cases act as plant growth-promoting fungi (Clocchiatti et al., 2020; Ozimek and Hanaka, 2021).These findings underscore that mulching can significantly elevate soil microbial abundance and optimize community structure, thereby fostering plant growth and improving agricultural productivity.

Mulching has a pronounced impact on the diversity and functional communities of rhizosphere microorganisms. In the FM treatment, there was a notable decrease in the abundance of Gram-positive bacteria, which showed a significant negative correlation with soil MC (**Supplementary Figures S3**). Under conditions of adequate water availability, the rhizosphere tends to be preferentially colonized by Gram-negative bacteria (Xu et al., 2018), which are typically predominant in fertile soils. The efficiency of Gram-negative bacteria in nutrient utilization is linked to their role in the production and accumulation of microbial residues. They contribute to the enhancement of soil organic carbon (SOC) in fertile soils by facilitating the turnover of microbial biomass (Fanin et al., 2019). The soil MCs in CK and SM treatments were significantly lower than that in FM (**Figure 2**), which corresponded with an increase in the abundance of Gram-positive bacteria, leading to accelerated SOM consumption. This pattern suggests that mulching influences soil health and plant growth by modifying the soil microbial community composition and selectively recruiting specific phenotypic microorganisms, thereby affecting the overall functionality of the soil ecosystem.

### 4.3 Mechanisms of pear yield and quality improvement

The co-occurrence network analysis revealed that specific bacterial groups responded similarly to a particular treatment, clustering together within the rhizosphere bacterial network. This pattern suggests that mulching significantly influences the bacterial ecological network (Dong et al., 2017; Tian et al., 2022), potentially by regulating rhizosphere bacterial community taxa that exhibit redundant functions. The network module results indicated that the FM treatment substantially enriched the phyla Proteobacteria and Acidobacteria. These phyla dominated in the ecological module M5 (**Figure 5B**), and their cumulative abundance was positively correlated with pear yield and quality (**Figures 5C, D**). Proteobacteria, Gemmatimonadetes and Acidobacteria, which are typical Gram-negative bacteria (**Figure 5E**), have been associated with increases in crop biomass (Bai et al., 2024; Shi et al., 2023) and enhancements in plant disease resistance (Purtschert-Montenegro et al., 2022). These groups have been reported to promote plant growth mainly through accelerating nutrient cycling, enhancing organic matter decomposition, and contributing to disease suppression. These findings underscore how mulching strategies can selectively enrich specific microbial taxa within the rhizosphere, which in turn contribute to various agricultural benefits, including enhanced yield, quality, and disease resistance in crops.

The findings of this study confirm that mulching effectively enhances both the yield and quality of pears, aligning with outcomes from previous research. SEM provided additional insights (**Figure 6**), illustrating that mulching modifies soil MC, which subsequently influences the ecological module—specifically enriching Gram-negative bacteria and altering the ecological network structure. These changes are pivotal in promoting fruit quality improvements typically associated with Gram-positive bacteria. The unique microbial community compositions and the increased relative abundances of specific microbial groups within modules, particularly M5, were instrumental in improving pear yield and quality. Therefore, the mechanisms through which mulching enhances pear yield and quality include direct effects on soil nutrients and MC, as well as indirect effects through the regulation of the abundance of Gram-negative bacteria within the rhizosphere microbial community and adjustments to the microbial ecological network structure. In conclusion, mulching regulates the abundance and diversity of specific microbial groups and fosters the growth of both the roots and above-ground parts of pear trees by modulating rhizosphere soil physicochemical properties. These comprehensive effects ultimately culminate in improved pear yield and quality.

## 5 Conclusions

In the arid region of Northwest China, where drip irrigation is prevalently utilized, plastic film mulching significantly enhances pear yield and quality compared to straw mulching. The beneficial effects of plastic film mulching on pear yield and quality are attributed to improvements in soil physicochemical properties, such as soil moisture content and organic matter. Additionally, this mulching method positively affects the soil ecosystem by enriching Gram-negative bacteria and altering microbial ecological modules. The integration of plastic film mulching with drip irrigation represents a promising technological approach for enhancing soil quality as well as fruit yield and quality in this arid region. This combination is particularly significant for agricultural production in arid areas, offering a strategic solution to optimize resource utilization and boost agricultural output efficiently.

## Data Availability

The datasets presented in this study can be found in online repositories. The names of the repository/repositories and accession number(s) can be found in the article/**Supplementary Material**.
