# Supplementary material for: Mulching influences pear yield and quality by changing rhizosphere microbial community structure in the arid region of Northwest China
Source: Front Plant Sci. 2025 Sep 29;16:1633540. doi: 10.3389/fpls.2025.1633540 (PMC12515920; doi:10.3389/fpls.2025.1633540)
Supplement: Supplementary file 1 [file Table1.docx]

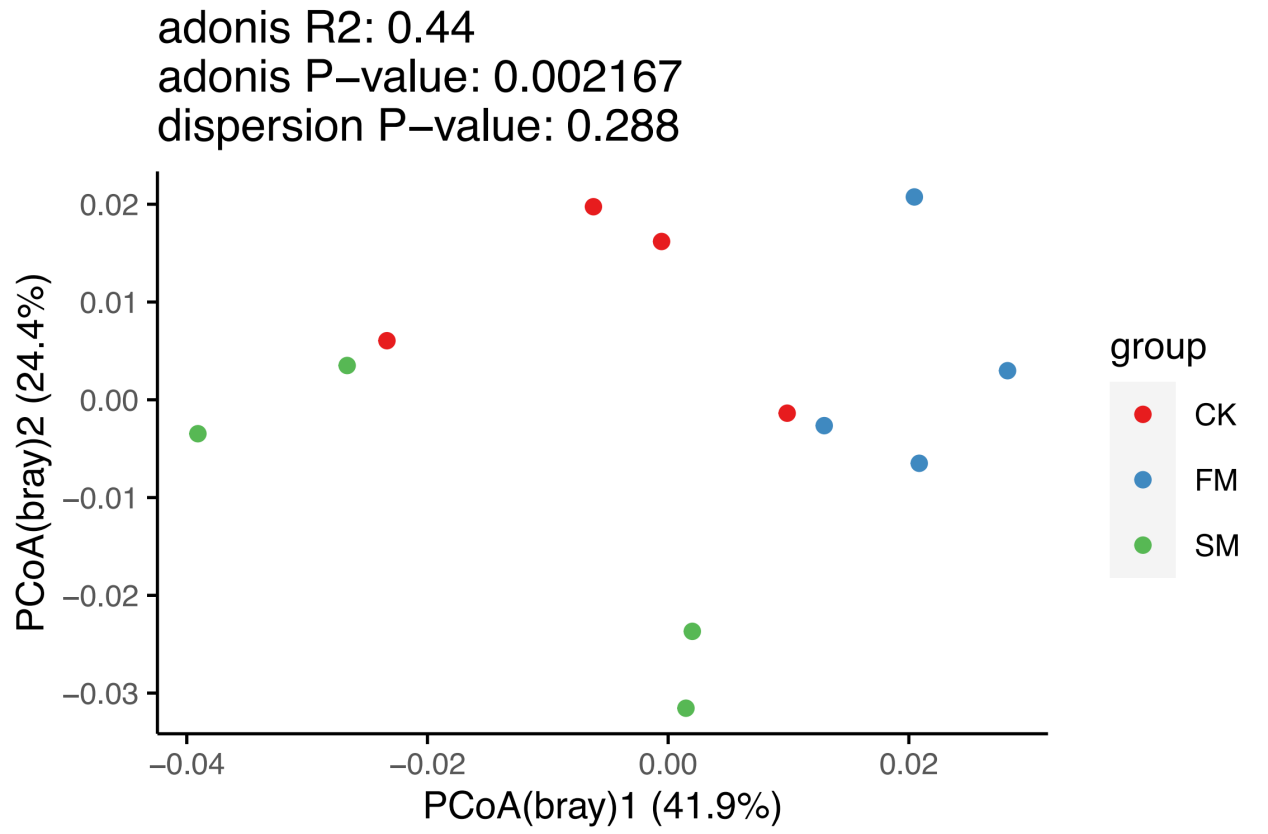


Figure S1 Fruit quality β-diversity (based on Bray-Curtis distance) in different treatments. CK, no mulching; FM, plastic film mulching; SM, straw mulching.


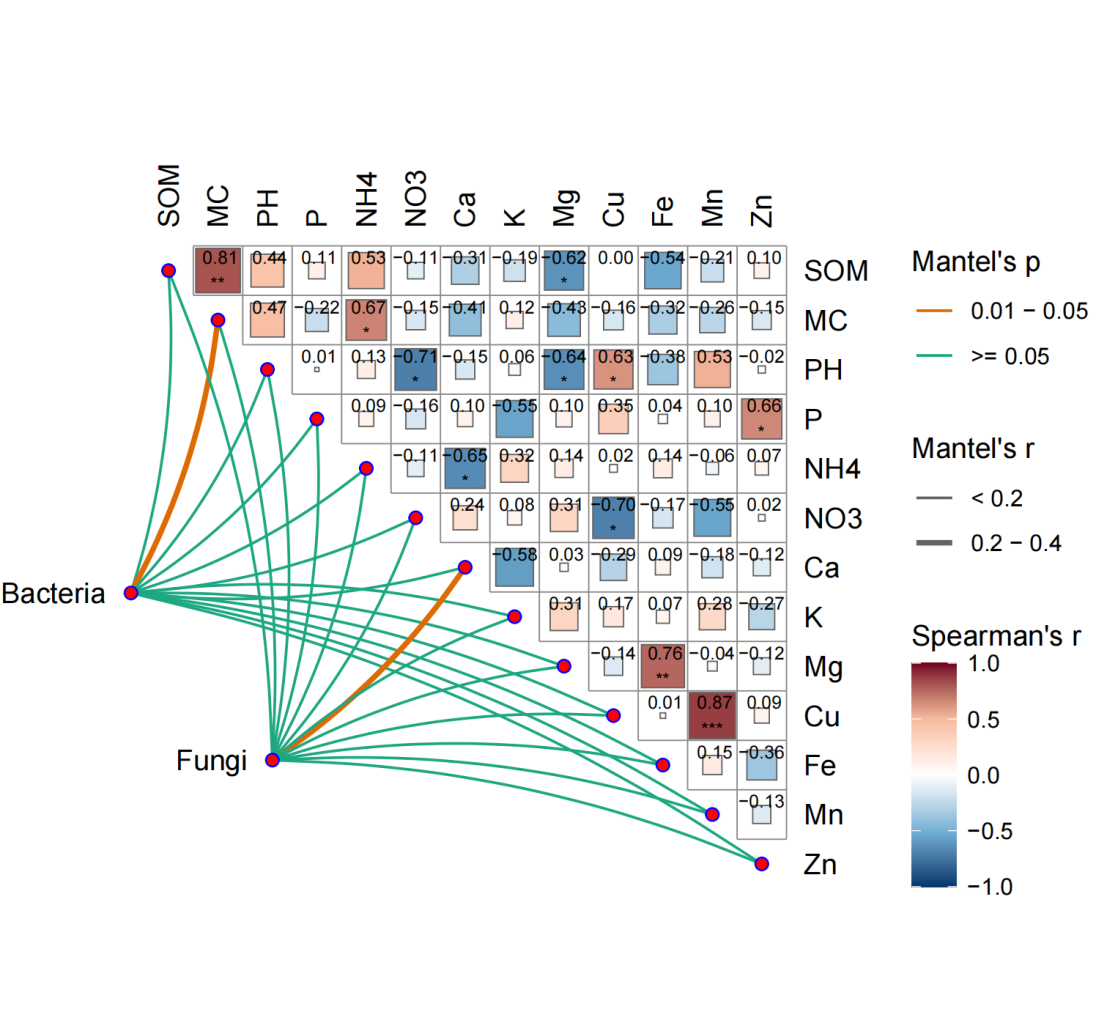
Figure S2 Environmental drivers of rhizosphere microbial community composition.****p* < 0.001; ***p* < 0.01; **p* < 0.05; ns; *p* > 0.05.


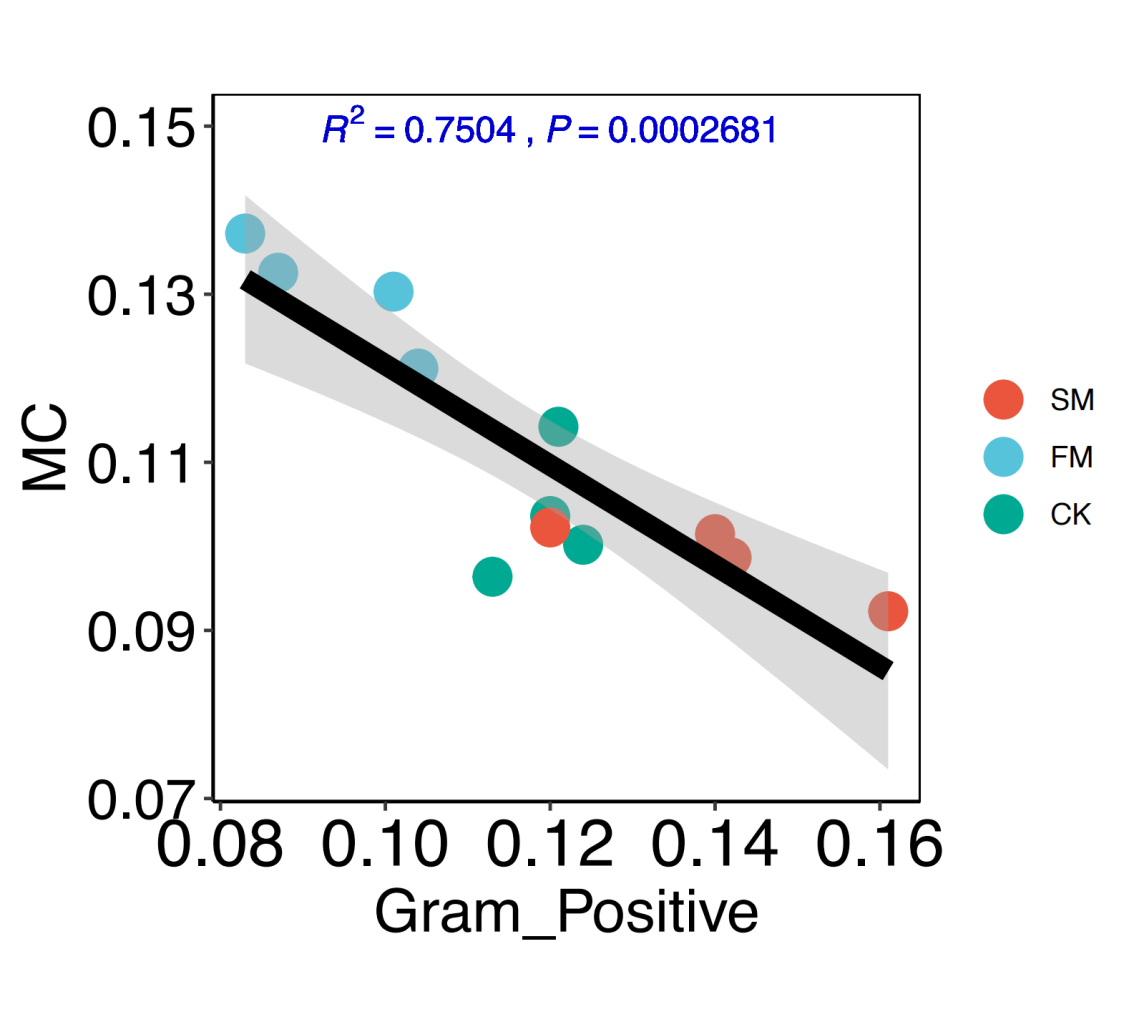


Figure S3 Significant correlation between soil water content and relative abundance of Gram-positive bacteria (P < 0.05). CK, no mulching; FM, plastic film mulching; SM, straw mulching.


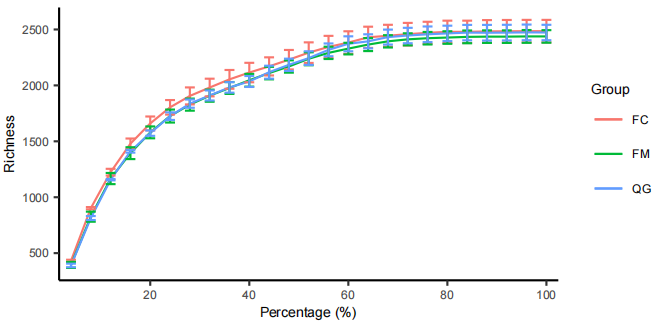

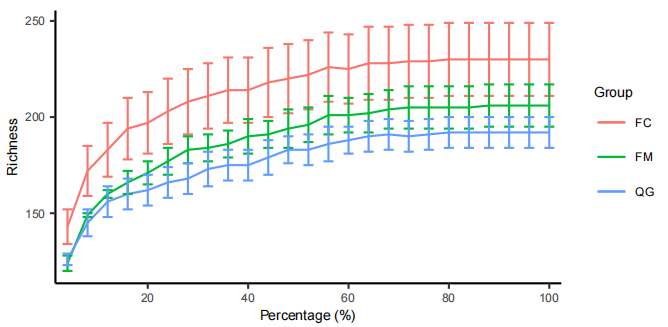


Figure S4. Rarefaction curves of bacterial 16S rRNA and fungal ITS rRNA ASVs across all samples. Curves approached saturation, indicating sufficient sequencing depth.
